# Protocorm-like-body extract of *Phalaenopsis aphrodite* combats watermelon fruit blotch disease

**DOI:** 10.3389/fpls.2022.1054586

**Published:** 2022-11-29

**Authors:** Bo-Lin Ho, Jhun-Chen Chen, Tzu-Pi Huang, Su-Chiung Fang

**Affiliations:** ^1^ Biotechnology Center in Southern Taiwan, Academia Sinica, Tainan, Taiwan; ^2^ Agricultural Biotechnology Research Center, Academia Sinica, Taipei, Taiwan; ^3^ Department of Plant Pathology, National Chung Hsing University, Taichung, Taiwan; ^4^ Innovation and Development Center of Sustainable Agriculture, National Chung Hsing University, Taichung, Taiwan; ^5^ Master’s and PhD Degree Program of Plant Health Care, Academy of Circular Economy, National Chung Hsing University, Nantou, Taiwan; ^6^ Biotechnology Center, National Chung Hsing University, Taichung, Taiwan

**Keywords:** *Acidovorax citrulli*, bacterial fruit blotch (BFB), protocorm-like-body, Phalaenopsis orchids, antibacterial activity

## Abstract

Bacterial fruit blotch, caused by the seedborne gram-negative bacterium *Acidovorax citrulli*, is one of the most destructive bacterial diseases of cucurbits (gourds) worldwide. Despite its prevalence, effective and reliable means to control bacterial fruit blotch remain limited. Transcriptomic analyses of tissue culture-based regeneration processes have revealed that organogenesis-associated cellular reprogramming is often associated with upregulation of stress- and defense-responsive genes. Yet, there is limited evidence supporting the notion that the reprogrammed cellular metabolism of the regenerated tissued confers *bona fide* antimicrobial activity. Here, we explored the anti-bacterial activity of protocorm-like-bodies (PLBs) of *Phalaenopsis aphrodite*. Encouragingly, we found that the PLB extract was potent in slowing growth of *A. citrulli*, reducing the number of bacteria attached to watermelon seeds, and alleviating disease symptoms of watermelon seedlings caused by *A. citrulli*. Because the anti-bacterial activity can be fractionated chemically, we predict that reprogrammed cellular activity during the PLB regeneration process produces metabolites with antibacterial activity. In conclusion, our data demonstrated the antibacterial activity in developing PLBs and revealed the potential of using orchid PLBs to discover chemicals to control bacterial fruit blotch disease.

## Introduction

Bacterial fruit blotch (BFB) is a serious seedborne pathogen for watermelon and melon worldwide and its outbreaks have caused severe fruit loss in the Americas, Asia, Europe, the Middle and Far East, and Australia (Burdman et al., 2005; Bahar and Burdman, 2010; Burdman and Walcott, 2012). In Taiwan, BFB disease was first reported in 1992-1993 (Tzeng et al., 2010). The recurrence of BFB in 1994 caused more than 60% loss of the watermelon crop nationwide (Tang, 1997; Cheng et al., 2000; Cheng, 2009; Tzeng et al., 2010). The primary source of inoculum is often contaminated seeds (Assouline et al., 1997). Because of the destructive nature of BFB disease, evaluation of pathogen contamination in seed lots prior to their sale and distribution has become a critical practice for seed companies. Based on the National Seed Health System USDA standard (http://www.seedhealth.org/cb1-1), one infected seedling in 30,000 tested seeds can be sufficient to lead to rejection of the entire seed lot. However, seed disinfestations and chemical control only have limited efficacy in controlling BFB (Burdman and Walcott, 2012). Even though seedling grow-out assay is widely used to evaluate seed health, infected seedlings may or may not show disease symptoms. It has been reported that the amount of bacteria present on a seed, environmental conditions, virulence levels of the strains, and the susceptibility of host plants as a whole affect BFB outbreaks (Schaad et al., 2003; Bahar and Burdman, 2010). If infected seedlings are not detected, they might be transplanted into the field and become the primary source of inoculum for field outbreaks. To date, no BFB disease resistant plants have been developed and BFB management remains a major challenge to global watermelon and melon agriculture (Bahar and Burdman, 2010; Islam et al., 2020). Hence, an innovative plan for a BFB management program is a pressing need.

The Orchidaceae represents one of the largest angiosperm families comprising more than 25,000 species that grow in wide range of habitats ranging from rainforest and mountain, to swamp and arctic tundra (Stokstad, 2015). Considering the rich diversity of the orchid species, it is likely that orchids provide a substantial resource of novel compounds for potential application. In fact, orchids have been utilized by humans for thousand years. For example, vanilla orchid *Vanilla planifolia*, probably endemic to tropical forests in Eastern Mexico, is a major source of vanilla (Bory et al., 2008). Additionally, orchids such as *Gastrodia elata*, *Bletilla striata*, and *Dendrobium* species have been used for medicinal purposes in China and other Asian countries for thousands of years (Bulpitt et al., 2007). Despite this background, only a few phytochemicals have been characterized from orchids and the potential of orchid derived-phytochemicals has not been fully explored.

Protocorm-like bodies (PLBs) morphologically resemble the orchid germinated structures, protocorms, but are derived from somatic explants *via* a *de novo* regeneration pathway (Jones and Tisserat, 1990; Chugh et al., 2009; Fang et al., 2022). Because each PLB has the ability to regenerate into an individual plant, PLB-based micropropagation is often used to produce clonal plantlets in the orchid industry (Yam and Arditti, 2009). Our previous comparative transcriptomic studies to dissect the developmental origin of PLB supported that PLB and protocorm share similar molecular signatures and unexpectedly revealed that many genes involved in plant defense responses are specifically enriched in the developing PLB (Fang et al., 2016; Fang, 2021; Fang et al., 2022). Considering the potential functions of PLB-enriched defense related genes, we explored the antimicrobial activity of PLB extract and tested its effects on BFB of watermelon. Our results demonstrate the antibacterial activity of PLB extract and suggest the potential of using the orchid PLBs for developing a reagent to control BFB.

## Materials and methods

### Pathogen

*Acidovorax citrulli* Aac153 (*A. citrulli* Aac153), isolated by the Laboratory of Phytopathogenic Bacteria, Department of Plant Pathology, National Chung Hsing University, Taiwan was a kind gift from Dr. Yi-Hsien Lin from National Pingtung University of Science and Technology. *A. citrulli* Aac153 has been shown to cause watermelon fruit blotch disease (Chang et al., 2019). *A. citrulli* Aac153 was stored at -75°C in tryptic soy broth (TSB) supplemented with 15% glycerol (v/v) and allowed to grow on selective medium AacG containing 0.5g/l KH_2_PO_4_, 2g/l Na_2_HPO_4_·12H_2_O, 2 g/l (NH_4_)_2_SO_4_, 5 g/l L-glutamic acid, 12.5 mg/l bromothymol blue, 15g/l agar, 20 mg/l ampicillin, 25 ppm/l cycloheximde as described previously (Chen, 2014). The single colony was used as inoculum for the primary culture. For subculture, single colony of primary culture was used to inoculate TSB and allowed to grow overnight at 28°C. The overnight culture was then grown on a selective AacG agar plate and the single colony was used as inoculum for the secondary culture. Only the primary and secondary cultures were used for inoculation in all the experiments. This strain produces reproducible, severe symptoms on the commercialized watermelon cultivar China Baby (Chang et al., 2019).

### PLB extraction

PLB tissues were homogenized by pestle and mortar in the presence of liquid nitrogen. One gram of pulverized PLB tissues was sonicated in the presence of 5 ml of ethyl acetate (EtOAc) for 30 mins (Branson 8510 DTH). The EtOAc-based PLB extract was incubated at 55°C for 10 min. Large tissue debris was removed by centrifugation at 3220 x g for 10 min. Supernatant was transferred to a new tube and concentrated by a rotary evaporator (EYELA, USA). The pellet was resuspended in 1 ml 100% MeOH. The PLB extract was then filtered by a 0.2 μm filter (13 mm Acrodisc Syringe filter, Pall) followed by concentration using a CentriVap Vacuum Concentrator (Labconco). The concentrated PLB-extract was flash frozen and stored at -80°C.

Frozen PLB extract was resuspended in 2 ml 30% MeOH. The 1 cc 50 mg Sep-PaK C18 cartridge (Waters) was first equilibrated with 1 ml of 100% MeOH followed by 1 ml of H_2_O once and then 1 ml of 30% MeOH once. For fractionation, solid phase extraction (SPE) was carried out by applying 1 ml PLB-based extract onto the equilibrated Sep-PaK C18 cartridge using a step gradient of MeOH-water mixture at a concentration of 30%, 45%, 60%, 80%, and 100% MeOH PLB and the eluents were collected individually. Methanol was allowed to evaporate by CentriVap Vacuum Concentrator (Labconco) and the fractionated PLB extract from 1 g tissues was pooled and resuspended in 100 μl 100% MeOH for seed infestation and pathogenicity assays as described below.

### Bacterial growth inhibition assay

Frozen PLB extract from 1 g of PLB tissues (see above) was resuspended directly in 1 ml 100% MeOH. The overnight *A. citrulli* Aac153 culture was pelleted by centrifugation and washed once with 5 ml TSB medium, and diluted to OD_600_ = ~0.05 with TSB medium. The diluted culture was mixed with crude PLB extract (933 μl bacterial culture + 67 μl PLB extract) and aliquoted into 6 technical replicates (100 μl each) to a 96-well microtiter plate and allowed to incubate at 28°C. The OD_600_ was recorded at 0, 15, 19 hours after incubation. For each biological replicate, the absorbance measurements of OD_600_ were recorded in three technical replicates. This experiment was repeated three times.

### Disease index scale

**Figure 1:**
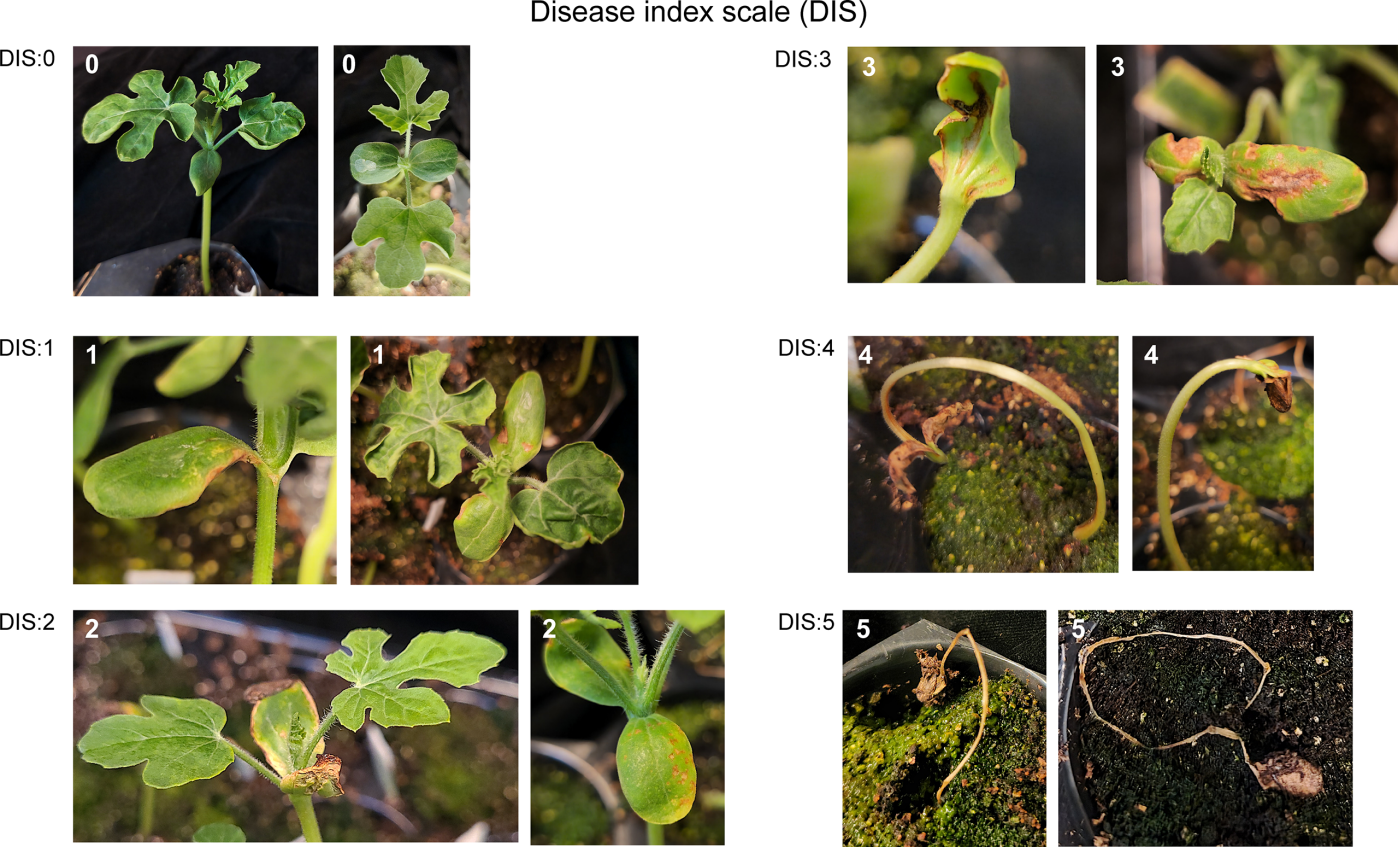
The represented disease severity index used to categorize disease symptoms of watermelon seedlings infected with *A. citrulli* Aac153. The numbers indicate the disease index described in Materials and Methods.

Disease symptoms of seedlings at 12 days after transplantation (DAT) were recorded. Normally, 12 DAT watermelon seedlings have two expanded true leaves. Disease index was rated as follows (**Figure 1**): 0, no symptoms; 1, slight (< 20%) water-soaking or necrotic spots on cotyledons or hypocotyls; 2, increased water-soaking or necrotic spots (>20%) on cotyledons or hypocotyls; 3, expanded water-soaking and necrosis (>50%) on cotyledons or hypocotyls, true leaves often failed to emerge from infected seedlings, for seedlings with emerging true leaves, leaves failed to expand and were often distorted; 4, bent seedlings with necrotic cotyledons and hypocotyls, no true leaves were observed; 5, falling-over seedlings with complete necrotic cotyledons and hypocotyls. Disease severity was calculated as DS (%) = [sum (class frequency × score of rating class)]/[(total number of plants) × (maximal disease index)] × 100.

### Seed infestation and pathogenicity assays

Watermelon seeds (China Baby) were purchased from Known-You Seed Company (Taiwan). For seed sterilization, seeds were imbibed in distilled water supplemented with 0.1% Triton X100 and gently shaken for 30 min. Imbibed seeds were sterilized by incubating with 75% ethanol for 5 min followed by washing with sterilized water 5 times. Seeds were allowed to dry in a laminar hood overnight.

For bacterial culture preparation, *A. citrulli* Aac153 culture that grew in 4 ml TSB overnight was pelleted by centrifugation at 3220 x g at 25°C for 10 min, washed once with 5 ml 0.5% carboxymethyl cellulose (CMC), resuspended in 0.5% CMC, and adjusted to OD_600_ to ~0.3. This preparation was used as the bacterial stock for seed infestation and seedling pathogenicity assays.

For seed infestation assay, five sterilized seeds were incubated with *A. citrulli* Aac153 culture in the presence (933 μl diluted bacterial culture + 67 μl fractionated PLB extract) or absence (933 μl diluted bacterial culture + 67 μl methanol) of PLB extract with gentle shaking (200 rpm) at 28°C for 2 h. The infested seeds were allowed to dry in a laminar flow hood overnight. The infected seeds were then resuspended in 1 ml AacG selective medium, incubated at 4°C for 30 min followed by incubation at 37°C for 1 h as described previously (Chen, 2014). Bacteria were concentrated by centrifugation at 15871 x g at 4°C for 10 min. The supernatant was carefully removed and the bacterial pellet was resuspended in 1 ml distilled water. The bacterial suspension was diluted 10 or 100 times by distilled water, plated on AacG selective plates, and allowed to grow at 28°C for three days. For each biological repeat, measurement of colony forming units was performed in three technical replicates. This experiment was repeated three times. A two-tailed Student’s t-test was applied. *, 0.05 > *p* > 0.01; **, *p* < 0.01. Only the treatments showing statistically significant reduction of bacteria were marked.

For seedling pathogenicity assay, the diluted *A. citrulli* Aac153 culture (OD_600_ to ~0.3) was further diluted 500 times with 0.5% CMC immediately before infection experiment. Eight sterilized seeds were incubated with 1 ml of diluted *A. citrulli* Aac153 culture in the presence (933µl diluted *A. citrulli* Aac153 + 67µl fractionated PLB extract, treatment) or absence (933 µl diluted *A. citrulli* Aac153 + 67µl MeOH, control) of PLB extract at 28°C with gentle shaking (200 rpm) for 24 h. The infested seeds were allowed to germinate in a humidity chamber at 32°C in the dark for 72 h. The germinated seedlings were then transferred to soil and allowed to grow in a growth chamber with a 16-h:8-h light:dark cycle under illumination of ~300 μmol photons m^-2^s^-1^ at 32°C. Plastic wrap was used to cover soil pots to maintain humidity and removed 5 days after transplantation. Disease symptoms were rated and recorded based on disease index scale (**Figure 1**) as described previously. The experiment was conducted three times. A two-tailed Student’s t-test was applied. **, 0.01 > *p* > 0.001; ***, *p* < 0.001.

### RNA extraction and RT-qPCR

RNA was extracted as described previously (Fang et al., 2016). Three micrograms of DNA-free RNA were reverse transcribed in the presence of a mixture of oligo(dT) and random primers in a 9:1 ratio using the GoScript Reverse Transcription System (Promega) based on the manufacturer’s instructions. Ten microliters of reverse transcription-PCR reaction contained 2.5 μL of 1:20 diluted cDNA, 0.2 μM of primers, and 5 μL of 2x KAPA SYBR FAST master mix (KAPA Biosystems). The amplification program was as follows: 95°C for 1 min, and 40 cycles at 95°C for 5 s and 58°C to 60°C for 20 s. PCR was performed in triplicate. Data are from technical triplicates and the error bars are presented as standard error of the mean. The RNA samples used for RT-qPCR analysis were independent from those for RNA-seq analyses. Primer pairs and the specified annealing temperature used for quantitative PCR are listed in **Supplementary Table 1**. *UBIQUITIN* was used as an internal control (Lin et al., 2014). The nomenclature of chalcone synthases was based on the previous study (Kuo et al., 2019). List of gene IDs used in this study and their corresponding IDs in the *P. aphrodite* databases are listed in **Supplementary Table 2**.

### Statistical analysis

All experiments were performed three times or as otherwise mentioned in the figure legends. The data are presented as means and standard deviations obtained from at least three replicates of a single experiment. The significant difference between the treatments was analyzed by running a Student’s t-test in IBM SPSS v.20.

## Results

### Plant defense-related genes are specifically upregulated in PLBs

Our previous RNA-seq study investigating the developmental origin of PLBs revealed that Gene Ontology (GO) terms such as oxidation-reduction process, terpene synthase activity, and stress responses are overrepresented in developing PLBs (Fang et al., 2016). The biochemical and biological properties of these GO terms are generally associated with plant defense responses (Field et al., 2006; Almagro et al., 2009; Ton et al., 2009; Gonzalez et al., 2010; Denance et al., 2013; Yang et al., 2013; Savatin et al., 2014). Among them, Phalaenopsis chalcone synthase (*CHS*) and flavonoid 3’ hydroxylase (*F3’ H*) genes, *PaCHS4*, *PaCHS5*, and *PaF3’ H1*, were preferentially upregulated in developing PLBs (**Table 1**). Chalcone synthase and flavonoid 3’ hydroxylase act at the initial steps to produce flavonoids- and isoflavonoid-type phytoalexins (Bak et al., 2011; Dao et al., 2011; Ahuja et al., 2012) that are part of plant defense responses (Ahuja et al., 2012). A PLB-enriched *PaCYC71A1*, which is related to Arabidopsis *CYP71A12* (Bak et al., 2011), encodes a putative cytochrome P450 monooxygenase (**Table 1**). Arabidopsis CYP71A12 takes part in biosynthesis of camalexin, a major phytoalexin important for disease resistance (Millet et al., 2010; Klein et al., 2013; Pastorczyk et al., 2020). Additionally, two PLB-enriched WRKY transcription factors, *PaWRKY3* and *PaWRKY4*, were identified. PaWRKY3 belongs to the group III WRKY transcription factors (**Supplementary Figure 1**) and is related to Arabidopsis WRKY70 (Wu et al., 2005). Arabidopsis *WRKY70* modulates salicylic acid (SA)- and jasmonic acid (JA)-mediated defense pathways to regulate plant immunity against bacterial pathogens (Li et al., 2004; Li et al., 2006; Zhou et al., 2018). *PaWRKY4*, on the other hand, encodes a group I WRKY transcription factor that is related to Arabidopsis *WRKY33* (**Supplementary Figure 1**), which is an important regulator for biosynthesis of camalexin and pathogen-associated molecular patterns (PAMP)/pathogen-triggered reactive oxygen species (ROS) and ethylene production (Qiu et al., 2008; Mao et al., 2011; Li et al., 2012; Zhao et al., 2020; Zhou et al., 2020).

**Table 1:** Plant defense-related genes are enriched in developing PLBs as shown by RNA-seq analysis.

		FPKM values										
Transcript ID	Annotation	30/40 DAP	50/60 DAP	70/80 DAP	90/100/120 DAP	140/160 DAP	180/200 DAP	PLB	Protocorm	Young leaves	Stalk buds	Floral stalks
orchid.id124284.tr400924	PaCHS4	1.7	0.6	0.2	0.4	2.0	2.3	185.9	10.7	1.4	0.8	0.9
orchid.id121282.tr400924	PaCHS5	0.6	0.4	0.1	0.3	2.3	2.2	186.2	15.1	1.3	0.5	0.6
orchid.id17741.tr406385	PaF3’ H1	7.56	9.43	25.19	11.91	5.96	10.07	194.62	5.43	7.90	6.39	4.89
orchid.id115099.tr56794	PaCYP71A1	0.2	0.1	0.1	0.7	1.9	1.4	395.4	23.6	0.1	0.2	0.1
orchid.id36575.tr215222	PaWRKY3	0.0	0.0	0.0	0.0	0.0	0.0	6.1	0.0	0.0	0.0	0.0
orchid.id184974.tr136611	PaWRKY4	0.0	0.0	0.0	0.0	0.0	0.0	9.1	1.1	0.0	0.0	0.0
orchid.id154271.tr406853	PaECR1	1.1	0.1	0.6	1.2	2.9	1.2	313.0	3.4	0.8	0.8	3.1
orchid.id163617.tr122100	PaPNP1	3.7	1.8	1.5	9.6	0.6	0.3	106.6	3.6	0.9	3.6	0.5
orchid.id156327.tr422593	PaRALF1	1.4	0.6	0.0	0.3	1.2	0.0	159.4	0.2	26.2	20.7	1.4
orchid.id133178.tr112803	PaMLP1	3.1	1.3	0.5	2.4	0.9	2.2	1046.2	7.6	0.2	7.1	6.9
orchid.id148348.tr112803	PaMLP2	4.3	1.4	0.6	3.1	0.9	2.6	1318.7	8.4	0.2	10.6	10.9
orchid.id136038.tr32844	PaPRX1	0.3	0.5	0.1	0.4	0.5	0.3	253.7	27.6	0.1	0.1	0.5
orchid.id123338.tr499847	PaCOMT1	1.4	1.1	1.4	3.0	4.0	3.7	645.1	28.4	1.8	15.0	8.7
orchid.id21743.tr69582	PaCOMT2	0.0	0.0	0.0	0.2	0.0	0.2	28.8	0.0	0.0	0.0	0.0

**Figure 2:**
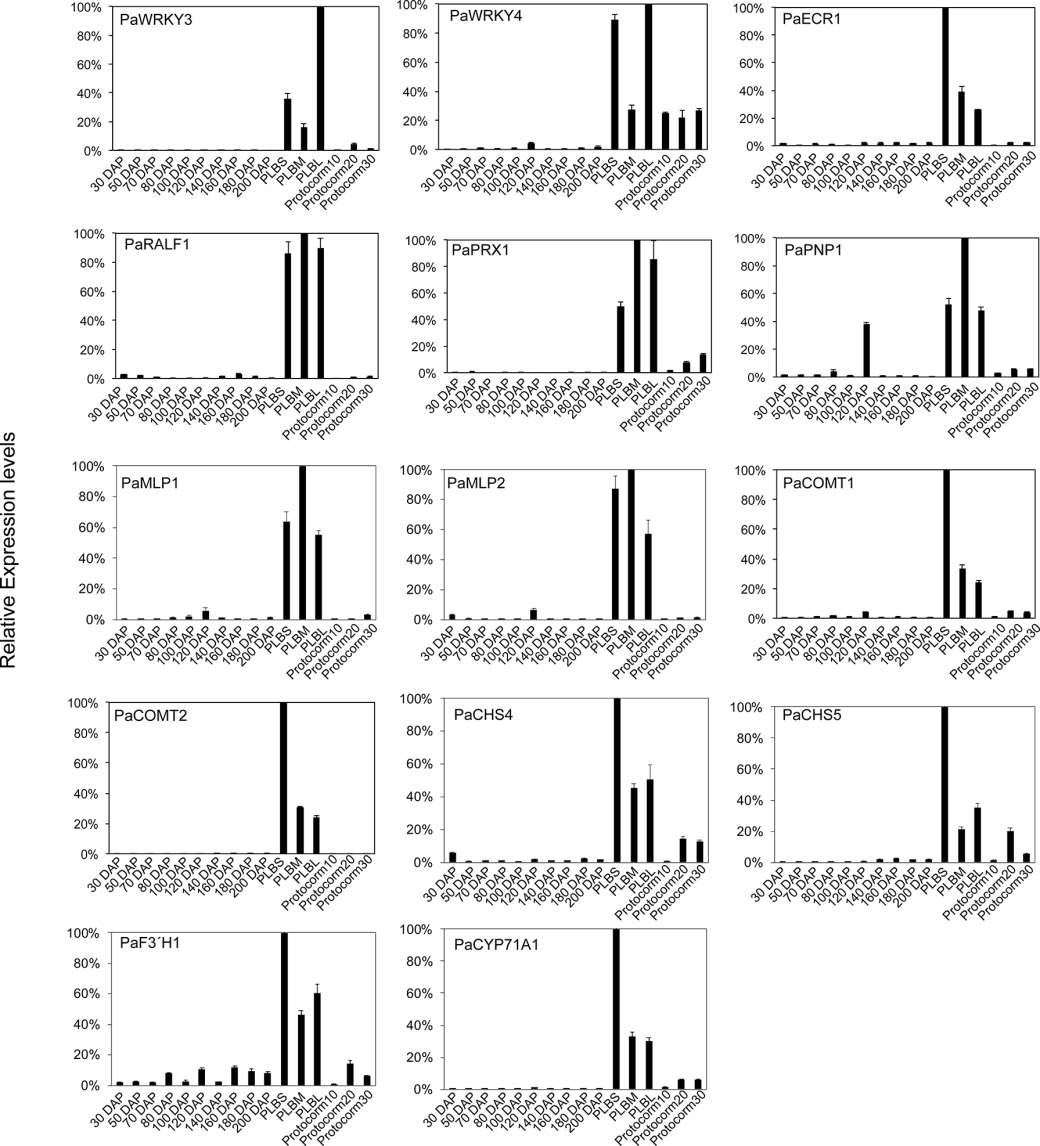
Expression profiles of the selected PLB-enriched genes in developing ovaries of *P. aphrodite* collected at 30 to 200 days after pollination (DAP), and developing PLBs and protocorms. Small PLBs (PLBS), medium PLBs (PLBM), large PLBs (PLBL), 10-d-old protocorms (protocorm10), 20-d-old protocorms (protocorm20), and 30-d-old protocorms (protocorm30) are defined as previously described (Fang et al., 2016) by RT-qPCR analysis. Expression was normalized to the Ubiquitin (*PaUBI*) signal. Data are from technical triplicates and the error bars are presented as standard error of the mean. Similar expression pattern was observed in RNA-seq data from two independently collected samples (Fang et al., 2016; Fang et al., 2022).

These PLB-enriched genes (**Table 1**) are also associated with other aspects of plant defense responses. For example, *PaECR1* encodes a potential enoyl-coA reductase that has been shown to play roles in plant defense responses in cotton and *P. amabilis* orchid (Fu et al., 2012; Mustafa et al., 2017). A potential PLANT NATRIURETIC PEPTIDE (PEP) encoded by *PaPEP1*A belongs to a family of peptides involved in regulation of defense responses and ion and water homeostasis (Gehring and Irving, 2013; Ficarra et al., 2018). *MAJOR LATEX PROTEIN1* (*PaMLP1*) and *NORCOCLAURINE SYNTHASE1* (*PaNCS1*) belong to pathogen-related 10 (PR10) and Bet v1 proteins (Radauer et al., 2008). MLP proteins are known to be involved in various abiotic and biotic responses (Yang et al., 2015; Wang et al., 2016; Holmquist et al., 2021). NCS proteins catalyze the first committed step of biosynthesis of benzylisoquinoline alkaloids (BIAs) that possess antimicrobial activity (Lee and Facchini, 2010). *PaRALF1*, on the other hand, encodes a protein that is related to Arabidopsis RAPID ALKALINIZATION FACTOR (RALF) peptides, which are found to interact with receptor-like kinase FERONIA to modulate ROS production and plant immune responses (Stegmann et al., 2017; Li et al., 2018; Abarca et al., 2021). The PLB-enriched genes, *PaCOMT1* and *PaCOMT2*, encode putative caffeic acid O-methyltransferases. Rice COMT is reported to have N-acetylserotonin O-methyltransferase (ASMT) activity that converts N-acetylserotonin to melatonin (Byeon et al., 2015), which modulates ROS and SA levels to improve plant responses to various abiotic and biotic stresses (Lee et al., 2015; Khan et al., 2020). Additionally, *PaPRX1* encodes a PLB-enriched peroxidase, which may take part in plant defense responses (O’Brien et al., 2012). The expression patterns of the described PLB-enriched genes have been documented in an independent RNA-seq dataset (Fang et al., 2022) and validated by RT-qPCR analysis in a separate set of samples (**Figure 2**).

### Crude PLB extract slows growth of *A. citrulli* Aac153

**Figure 3:**
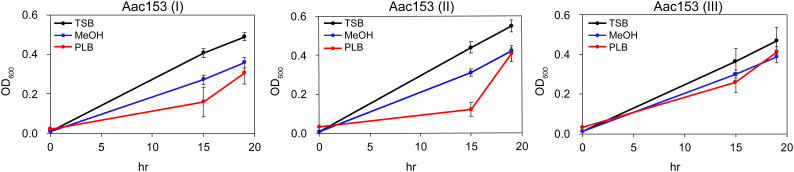
Crude PLB extract affected growth of *A. citrulli* Aac153. Aac153, *A. citrulli* Aac153 culture. TSB, cells were allowed to grow in TSB medium. MeOH, cells were allowed to grow in TSB medium containing 6.7% MeOH. PLB, cells were allowed to grow in TSB medium supplemented with 6.7% PLB extract. hr, hour after incubation. I, II, and III represent three independent experiments.

Because many plant defense-relates genes were specifically induced in developing PLBs, we hypothesized that the dynamic metabolomic reprogramming of developing PLBs leads to synthesis of antimicrobial metabolites. Ethyl acetate (EtOAc), which is commonly used for plant metabolite extraction (Tamokou et al., 2012; Oliveira et al., 2013; Lien et al., 2014; Yang et al., 2022), was used to prepare PLB crude extract. The crude extract was then tested for its effect on growth of three plant pathogens including *Acidovorax citrulli* Aac153 (watermelon bacterial fruit blotch disease), *Pectobacterium carotovorum* subsp. *carotovorum* (bacterial soft rot disease), and *Xanthomonas citri* pv. mangiferaeindicae (mango bacterial black spot disease). Because PLB crude extract showed consistent inhibitory effect on growth of *A. citrulli* Aac153 in the preliminary test (data not shown), we decided to focus on *A. citrulli* Aac153. To confirm this inhibitory effect, bacterial growth was monitored over 19 hours. In three separate experiments, PLB crude extract slowed the growth of *A. citrulli* Aac153 15 hours after treatment (**Figure 3**), indicating the presence of anti-bacterial activity in the developing PLBs. However, growth of *A. citrulli* Aac153 eventually caught up 19 hours after incubation, suggesting other substances may interfere with the inhibitory activity.

### PLB extract reduces number of *A. citrulli* Aac153 bacteria associated with watermelon seeds

**Figure 4:**
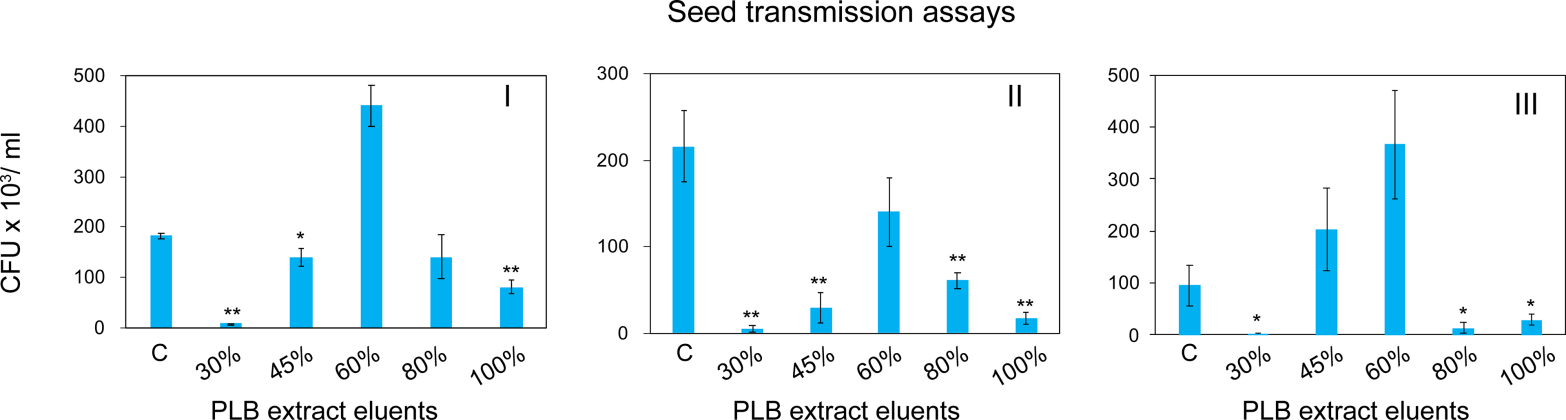
Watermelon seed infestation assay. CFU, colony forming unit. CFU Data were collected from 5 pooled watermelon seeds. C, no PLB extract control. 30%, PLB extract eluted by 30% MeOH-H2O. 45%, PLB extract eluted by 45% MeOH-H2O. 60%, PLB extract eluted by 60% MeOH-H2O. 80%, PLB extract eluted by 80% MeOH-H2O. 100%, PLB extract eluted by 100% MeOH-H2O. The experiment was repeated three times with similar results. I, II, and III represent three independent experiment. A two-tailed Student’s t-test was applied. *0.05 > *p* > 0.01; **p < 0.01. Only the treatments showing statistically significant reduction of bacteria were marked.

To enrich and separate the active metabolites from interfering substances, PLB crude extract was fractionated by solid phase extraction (SPE) based on the chemical polarity. Chemicals eluted with different concentrations of MeOH were collected for seed infestation test. Watermelon seeds were inoculated with *A. citrulli* Aac153 in the presence of different PLB eluents (see Materials and Methods). Interestingly, 30% MeOH PLB eluent was shown to be most effective in reducing the number of bacteria associated with watermelon seeds (**Figure 4**). Only 2.1% to 6.5% of bacterial cells remained after co-incubation with 30% MeOH-water PLB eluent. Co-incubating seeds with the 100% MeOH PLB eluents was also effective in reducing bacterial number but to a lesser extent (between 7.9% to 53.2%) with relatively large variations as compared to the 30% MeOH-water PLB eluents. This indicates that compounds with the potent antibacterial activity were enriched in 30% MeOH-water PLB eluent.

### PLB extract alleviates disease symptoms caused by *A. citrulli* Aac153

Because 30% MeOH PLB eluent was effective in reducing the number of bacteria attached to the watermelon seeds, we then investigated whether it can protect watermelon seedlings from *A. citrulli* Aac153 infection. To this end, watermelon seeds were incubated with *A. citrulli* Aac153 culture in the presence of 1x or 1/5x of 30% MeOH PLB eluent.

**Figure 5:**
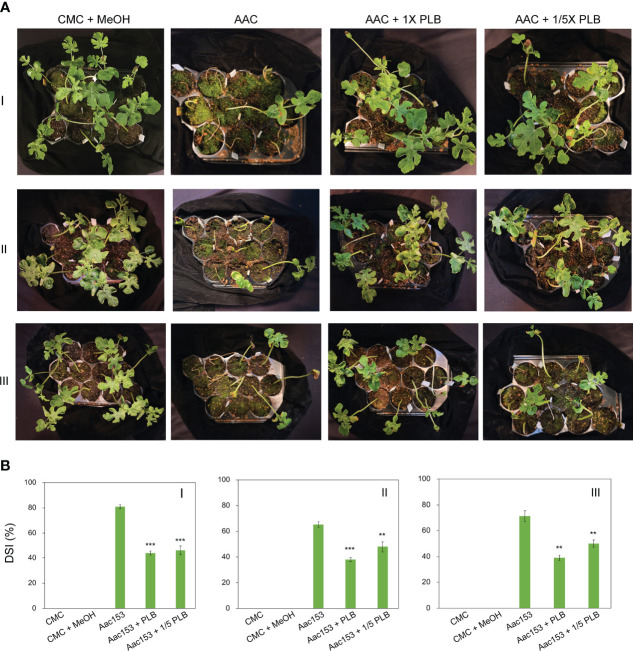
Thirty percent MeOH PLB eluent alleviates disease symptoms of watermelon bacterial blotch disease. **(A)** Disease symptoms of *A citrulli* Aac153-infested seedlings at 12 days after transplantion (DAT). **(B)** Disease severity index (DSI) of 12 DAT watermelon seedlings after treatments. CMC + MeOH, seeds were incubated with 6.7% MeOH in CMC medium. Aac153, seeds were incubated with *A citrulli* Aac153 in the presence of 6.7% MeOH. Aac153 + PLB, seeds were incubated with *A citrulli* Aac153 in the presence of 30% MeOH PLB extract. Aac153 + 1/5 PLB, seeds were incubated with *A citrulli* Aac153 in the presence of one-fifth 30% MeOH PLB extract. The experiment was repeated three times. I, II, and III represent three independent experiments. Twenty-two seeds were used in each experiment except CMC and CMC + MeOH treatments. A two-tailed Student’s t-test was applied. **0.01 > *p* > 0.001; ***p < 0.001.

Watermelon seedlings from seeds incubated with CMC or CMC + 6.7% MeOH were used as controls, and no disease symptoms were observed on these seedlings (**Figure 5A**; **Supplementary Figure 2**). On the other hand, watermelon seedlings inoculated with *A. citrulli* Aac153 inoculation, showed water-soaking spots and necrotic lesions on the hypocotyl or cotyledons, typical BFB symptoms (Walcott, 2008; Bahar and Burdman, 2010), at 12 days after transplantation (DAP). Co-treatment of 1x 30% MeOH PLB eluent alleviated disease symptoms on the *A. citrulli* Aac153-infected seedlings. Moreover, 1/5 x 30% MeOH PLB eluent was also potent in protecting the *A. citrulli* Aac153-infected seedlings. This experiment was conducted three times and similar results were obtained each time. Disease assessment was quantified by the disease index scale as detailed in Materials and Methods. Disease severity of the *A. citrulli* Aac153-infected seedlings ranged from 65.5% to 81% (**Figure 5B**). The disease severity of the *A. citrulli* Aac153-infected seedlings treated with 1x 30% MeOH PLB eluent was reduced to 38.0% to 46%. Even 1/5 x 30% MeOH PLB eluent was able to protect the *A. citrulli* Aac153-infected seedlings and disease severity was reduced to 46% to 48.2%. Importantly, PLB treatment at the higher concentration only slightly affected seed germination (*p* value = 0.03, **Table 2**). Together, we conclude that PLB-derived metabolites possess the antibacterial activity that protects watermelon seedlings from *A. citrulli* Aac153 infection.

**Table 2:** Germination rate of watermelon seeds.

	CMC	CMC + MeOH	Aac153	Aac153 + PLB	Aac153 + 1/5 PLB
Germination rate	100.0 ± 0.0%	90.0 ± 9.1%	87.9 ± 8.0%	89.4 ± 6.1%	93.9 ± 3.0%
*p* value	N/A	0.42	0.15	0.03	0.18

CMC, seeds incubated with CMC medium before germination. CMC + MeOH, seeds incubated with 6.7% MeOH in CMC medium before germination. Aac153, seeds incubated with A. citrulli Aac153 in the presence of 6.7% MeOH. Aac153 + PLB, seeds incubated with A. citrulli Aac153 in the presence of 30% MeOH PLB extract. Aac153 + 1/5 PLB, seeds incubated with A. citrulli Aac153 in the presence of one-fifth 30% MeOH PLB extract. Twenty-two seeds were used in each experiment except CMC and CMC + MeOH control experiments. For CMC and CMC + MeOH treatments, eleven seeds were used in each experiment. The experiment was repeated three times. N/A, not applicable. p values were derived from SPSS Student’s t-test analysis.

## Discussion

### Phalaenopsis orchid PLBs possess the antibacterial activity

Accumulated studies have provided molecular evidence linking pluripotency acquisition of plant regeneration processes to activation of defense responses (Chupeau et al., 2013; Ikeuchi et al., 2017; Iwase et al., 2021; Li et al., 2021). Furthermore, defense- or stress-associated pathways are proposed to be part of a gene regulatory network for cell proliferation and organ regeneration (Heyman et al., 2018; Wu et al., 2020a; Zeng et al., 2021). However, there is little evidence to support the notion that the rewired gene regulatory network of regenerated tissues is capable of synthesizing antimicrobial metabolites. Here, we showed that orchid PLB contains antibacterial substances that are potent in slowing the growth of *A. citrulli* Aac153, reducing the number of *A. citrulli* Aac153 associated with watermelon seeds, and protecting watermelon seedlings from severe infection by *A. citrulli* Aac153. Why would the developing PLBs possess the antibacterial activity? PLB is a regenerated structure induced by cutting during tissue culture (Yam and Arditti, 2009). Tissue injury caused by cutting during tissue culture triggers wounding-induced responses that mimic mechanical wounding triggered by herbivores and insects. It is therefore possible that wounding activates responses that contribute to production of antibacterial activity in PLBs. Wounding induced by herbivores and insects is known to trigger *de novo* synthesis of ethylene, jasmonic acid, and abscisic acid (Hyodo et al., 1983; Peña-Cortés et al., 1995; Bergey et al., 1996; Bouquin et al., 1997) that subsequently induce plant immunity responses to protect plants from infection by microbial pathogens (Savatin et al., 2014). Wounding also induces an array of immunity-related transcription factors such as WRKY transcription factors whose functions are to activate plant defense responses and prevent bacterial and fungal infection (Li et al., 2004; Li et al., 2006; Zheng et al., 2006; Pandey et al., 2010; Sarris et al., 2015; Zhou et al., 2018). Coincidently, some of the WRKY transcription factors also play a role in tissues regeneration (Che et al., 2006; Xu et al., 2012; Iwase et al., 2021). In this study, we showed that *PaWRKY3* and *PaWRKY4* (**Table 1**, **Figure 2**) are PLB-enriched transcription factors. *PaWRKY3* and *PaWRKY4* are the homologs of Arabidopsis defense response regulators, *WRKY70* and *WRKY33*, respectively (Li et al., 2017; Zhou et al., 2018). Arabidopsis *WRKY70* is directly regulated by the key immunity signaling regulator NONEXPRESSOR OF PR GENES1 (NPR1) and *wrky70* mutant displayed reduced resistance to the oomycte *Hyaloperonospora parasitica* (Wang et al., 2006; Knoth et al., 2007). Arabidopsis *WRKY33*, on the other hand, is required for pathogen-induced biosynthesis of camalexin and ethylene response (Mao et al., 2011; Li et al., 2012). Considering the antimicrobial activity of PLB extract, we hypothesize that *PaWRKY3* and *PaWRKY4* may be part of cell reprogramming networks in developing PLBs that contribute to the defense activation and accumulation of antimicrobial metabolites. The functions of these *PaWRKY3* and *PaWRKY4* transcription factors remain to be determined.

Phytoalexins are reported to play important roles in combating a broad range of bacterial and fungal pathogens (Glazebrook and Ausubel, 1994; Graham et al., 2007; Ahuja et al., 2012; Schmelz et al., 2014). Because wounding has been reported to induce phytoalexin biosynthesis (Guillet and De Luca, 2005; Naoumkina et al., 2007; Farag et al., 2008), we speculated that phytoalexins make up part of the PLB-based antibacterial metabolites. Since chalcone synthases (*PaCHS4* and *PaCHS5*) and flavonoid 3’ hydroxylase (*PaF3’ H1*) were specifically upregulated in PLBs, it is possible that flavonoids- and isoflavonoid-type phytoalexins were accumulated to provide the antimicrobial activity. However, we cannot exclude the possibility that other types of phytoalexins or cellular processes provide the inhibitory effect against *A. citrulli* Aac153. The active substance(s) remain to be purified and identified.

### Orchid PLBs may enable discovery of novel antimicrobial metabolites

Plants possess a rich repertoire of phytochemicals that are important for plants to combat pathogens and predators, and adapt to biotic and abiotic stresses in the natural environment (Pichersky and Lewinsohn, 2011; Moghe and Last, 2015). The fact that the stress-activated plant regeneration program is often associated with defense-associated cellular activity suggests that the molecular wiring of development and plant immunity processes is overlapping. This notion is supported by a recent study showing that plant development and immunity share a signaling network (Wu et al., 2020b). In addition to a signaling network, we hypothesize that the cellular metabolism is also reprogrammed to accommodate stress-associated organogenesis and plant immunity functions. The available genome and transcriptome databases of *P. aphrodite* (Chao et al., 2017; Chao et al., 2018) provide the molecular basis to discover pathways and to decode the molecular wiring of PLB-associated antimicrobial metabolites (Owen et al., 2017). We suggest that PLBs may be used as a metabolite tap for identifying novel antibacterial compounds. Identification and characterization of the PLB-associated antimicrobial metabolites may provide a new route to harness the chemical diversity of orchid species.

Bacterial fruit blotch disease is a serious threat to the cucurbit (gourd) industry. Even though seed sanitation with hydrochloric acid or peroxyacetic acid have been proven to be effective at eradicating pathogens from infested seeds, seed quality is affected substantially (Hopkins et al., 1996; Hopkins et al., 2003). Moreover, seed disinfestation treatments and chemical control in the field are limited in their ability to reduce the yield losses associated with BFB (Burdman and Walcott, 2012), most likely because the applied chemicals cannot reach bacteria that are associated with developing embryos and seed coats (Rane and Latin, 1992; Hopkins and Thompson, 2002; Dutta et al., 2016). Since PLB-based extract did not affect the viability or germination rate of the watermelon seeds (**Table 2**), it may serve as an alternative to control watermelon fruit blotch disease.

## Data availability statement

The original contributions presented in the study are included in the article/**Supplementary Materials**. Further inquiries can be directed to the corresponding authors.

## Author contributions

S-CF conceived the idea and coordinated the study. S-CF, B-LH, and T-PH designed the experiments. S-CF, B-LH, and T-PH analyzed the data. S-CF and T-PH wrote the manuscript. B-LH and J-CC conducted the experiments. All the authors have read and approved the final manuscript.

## Funding

This work was supported by the Ministry of Science and Technology (MOST 106-2313-B- 001-004-MY3 and MOST 109-2313-B-001 -015 -MY3 to SCF).

## Acknowledgments

We thank Dr. Yi-Hsien Lin for providing us *Acidovorax citrulli* Aac153 strain, Mr. Fu-Chieh Yang for technical support, and Ms. Miranda Loney for English editing.

## Conflict of interest

S-CF and B-LH are named inventors on a patent application pertaining to the technology that was filed by Academia Sinica.

The remaining authors declare that the research was conducted in the absence of any commercial or financial relationships that could be construed as a potential conflict of interest.

## Publisher’s note

All claims expressed in this article are solely those of the authors and do not necessarily represent those of their affiliated organizations, or those of the publisher, the editors and the reviewers. Any product that may be evaluated in this article, or claim that may be made by its manufacturer, is not guaranteed or endorsed by the publisher.
