# Supplementary material for: Protocorm-like-body extract of Phalaenopsis aphrodite combats watermelon fruit blotch disease
Source: Front Plant Sci. 2022 Nov 29;13:1054586. doi: 10.3389/fpls.2022.1054586 (PMC9745142; doi:10.3389/fpls.2022.1054586)
Supplement: Supplementary file 1 [file DataSheet_1.pdf]

Supplementary Table 1 List of primers used for RT-qPCR.

Gene name	Forward primer	Reverse primer	Amplicon size (bp)	Annealing temp (°C)
PaCHS4	5′-ACGGTCGAAACTGTCGTTCT-3′	5′-AATAGCCGCAAGCCTTAACA-3′	148 bp	58
PaCHS5	5′-TCGAAACTGTCGTTCTCCAA-3′	5′-TCCCCTTGAACCAATGAAAA-3′	142 bp	58
PaF3′ H1	5′-GGACGGCATAATGATCTGCT-3′	5′-CGAATTCGATTGTGTTCGAC-3′	151 bp	58
PaCYP71A1	5′-GCCCTCACAAGGAATCACAT-3′	5′-TCTGCCCCATTAGCAACTCT-3′	160 bp	58
PaWRKY3	5′-CTCAAATGGGAGGAATGGAC-3′	5′-TGTTGCACGACATCCTTGTT-3′	196 bp	58
PaWRKY4	5′-CCACAACGCACAAGAAAGAA-3′	5′-CGTCACCAAATGAAGCAGAA-3′	148 bp	58
PaECR1	5′-GCTGCAATGGAAGATTGGTT-3′	5′-TCGCGCAAGTCACAAAATTA-3′	152 bp	58
PaPNP1	5′-ACATGTACAGGTGCGACCAA-3′	5′-GCCGGCATTTAAATCTGCTA-3′	153 bp	58
PaRALF1	5′-TAGCGATGCAGTCATGGAAG-3′	5′-ACTTATGTGGTTGCCCGTCT-3′	133 bp	58
PaMLP1	5′-TTAAAACTCTGATAATTTGCTGGTCA-3′	5′-TTTGTAGCAAGCATAAATTACAACA-3′	150 bp	60
PaMLP2	5′-TGCTGATCAGTAAGAGTGTGTTTG-3′	5′-TCCTTTTAATTTTATTTATTCCGAACT-3′	124 bp	58
PaPRX1	5′-AAAAACGCAATCCCAAACAG-3′	5′-CCAGTTTGGTCCTCCAAGAG-3′	159 bp	58
PaCOMT1	5′-AGTGGGTTCTCCATGACTGG-3′	5′-CGTGCAAGATGCAGTGAGTT-3′	153 bp	58
PaCOMT2	5′-ATGTTGGTGGGGATGTGTTT-3′	5′-ACCTTCCCTCTGTTCGGAAT-3′	139 bp	60
PaUBI	5′-AACTCCATCGCCTTCCTCTT-3′	5′-TGAAGCATGGCATCAATTTC-3′	101 bp	58 to 60

Supplementary Table 2 List of gene IDs used in this study and their corresponding IDs in the *P. aphrodite* databases.
NA, not applicable.

Gene name	2016 Transcriptome Used in this study (Fang et al., 2016)	2022 Transcriptome (Fang et al., 2022)	Orchidstra 2.0 ID (Chao et al., 2018)
PaCHS4	orchid.id124284.tr400924	denovo.id119989.tr=219359	PAXXG122400
PaCHS5	orchid.id121282.tr400924	denovo.id88234.tr=400924	PAXXG122380
PaF3' H1	orchid.id17741.tr406385	denovo.id110376.tr=406385	PAXXG079820
PaCYP71A1	orchid.id115099.tr56794	denovo.id92994.tr=56794	PAXXG184140
PaWRKY3	orchid.id36575.tr215222	denovo.id125027.tr=316138	PAXXG318610
PaWRKY4	orchid.id184974.tr136611	denovo.id118424.tr=197162	PAXXG245730
PaECR1	orchid.id154271.tr406853	denovo.id113634.tr=130979	PAXXG077920
PaPNP1	orchid.id163617.tr122100	denovo.id136640.tr=64	PAXXG019080
PaRALF1	orchid.id156327.tr422593	denovo.id58873.tr=422593	NA
PaMLP1	orchid.id133178.tr112803	denovo.id128908.tr=398843	PAXXG159850
PaMLP2	orchid.id148348.tr112803	denovo.id65820.tr=112803	PAXXG159860
PaPRX1	orchid.id136038.tr32844	denovo.id76319.tr=32844	PAXXG036970
PaCOMT1	orchid.id123338.tr499847	denovo.id86615.tr=499847	PAXXG228500
PaCOMT2	orchid.id21743.tr69582	denovo.id133620.tr=53755	PAXXG228510

References:

Chao, Y.T., Chen, W.C., Chen, C.Y., Ho, H.Y., Yeh, C.H., Kuo, Y.T., et al. (2018). Chromosome-level assembly, genetic and physical mapping of *Phalaenopsis aphrodite* genome provides new insights into species adaptation and resources for orchid breeding. *Plant Biotechnol J* 16(12)**,** 2027-2041. doi: 10.1111/pbi.12936.

Fang, S.C., Chen, J.C., Chang, P.Y., and Lin, H.Y. (2022). Co-option of the *SHOOT MERISTEMLESS* network regulates protocorm-like body development in Phalaenopsis aphrodite. *Plant Physiol*. doi: 10.1093/plphys/kiac100.

Fang, S.C., Chen, J.C., and Wei, M.J. (2016). Protocorms and protocorm-like bodies are molecularly distinct from zygotic embryonic tissues in *Phalaenopsis aphrodite*. *Plant Physiol* 171(4)**,** 2682-2700. doi: 10.1104/pp.16.00841.

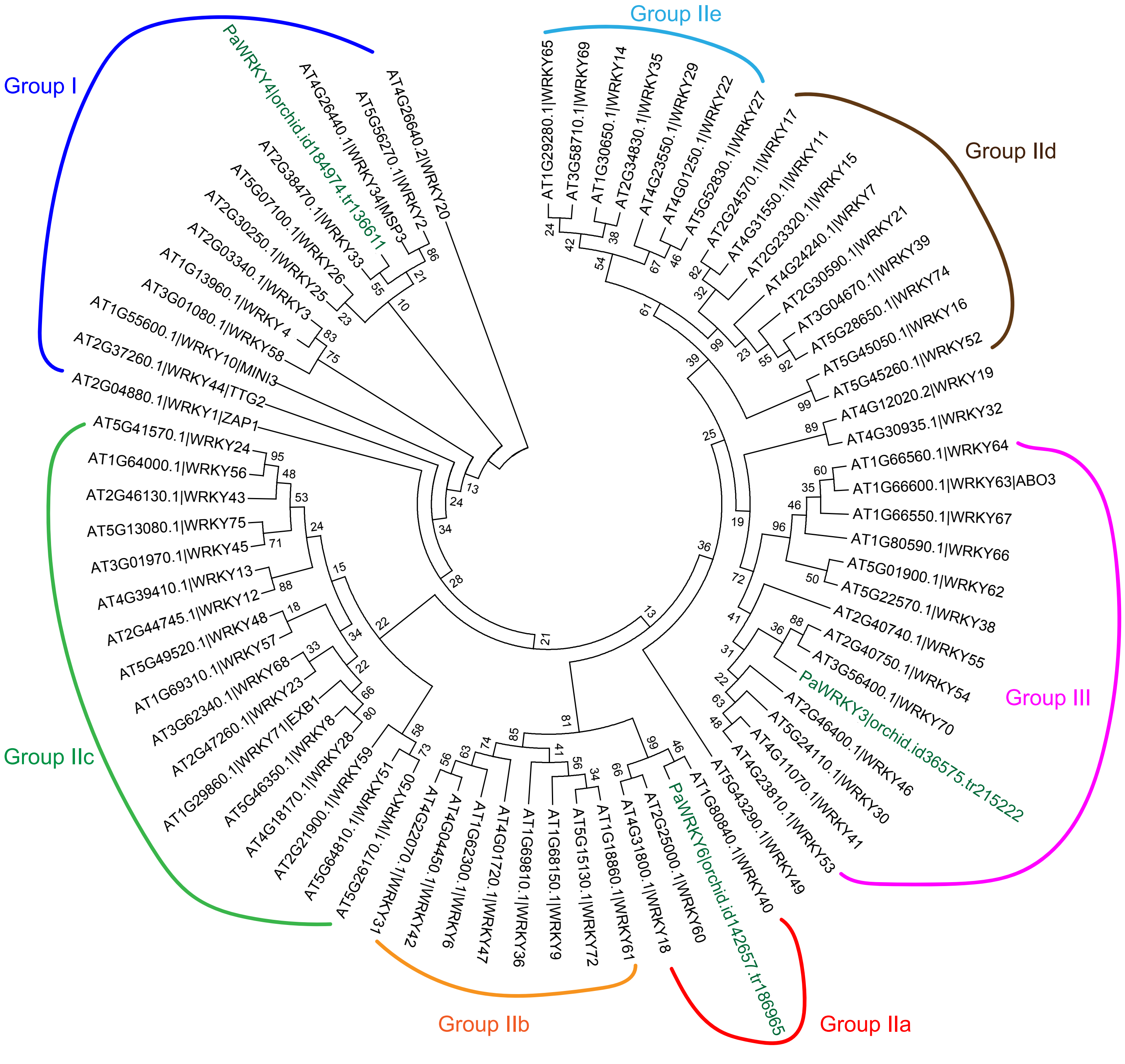

Supplementary Figure 1. Phylogenetic relationship of WRKY transcription factors of *Arabidopsis thaliana* (AT) and selected members from *Phalaenopsis aphrodite* (Pa). Full-length WRKY protein sequences were aligned by MUSCLE algorithm and the unrooted Maximum-likelihood tree was constructed with 1,000 bootstrap iterations. This figure was modified based on the WRKY tree in Zhang and Wang, 2005 (BMC Evol BIol).

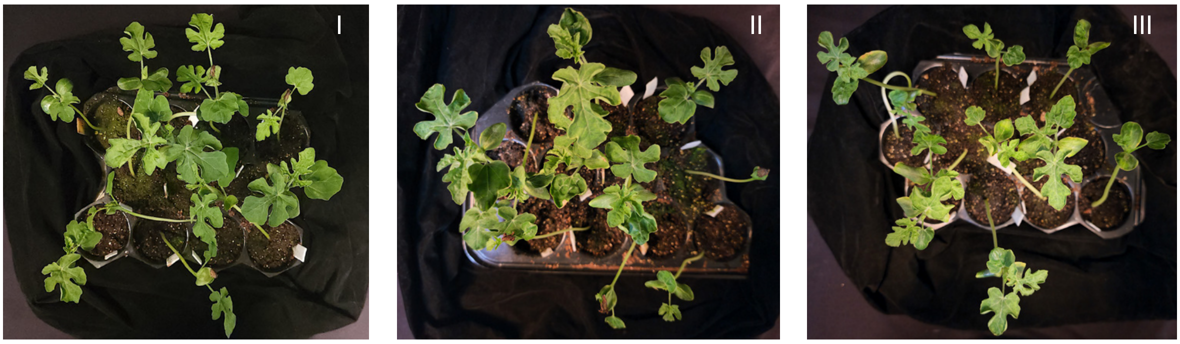

Supplementary Figure 2. No disease symptom was observed on watermelon seedlings from seeds incubated with CMC medium. The experiment was repeated three times. I, II, and III represent three independent experiment.
